# Preprinting is positively associated with early career researcher status in ecology and evolution

**DOI:** 10.1002/ece3.8106

**Published:** 2021-09-21

**Authors:** Jesse F. Wolf, Layla MacKay, Sarah E. Haworth, Marie‐Laurence Cossette, Morgan N. Dedato, Kiana B. Young, Colin I. Elliott, Rebekah A. Oomen

**Affiliations:** ^1^ Department of Environmental and Life Sciences Trent University Peterborough ON Canada; ^2^ Department of Forensic Science Trent University Peterborough ON Canada; ^3^ Department of Biosciences Centre for Ecological and Evolutionary Synthesis University of Oslo Oslo Norway; ^4^ Department of Natural Sciences Centre for Coastal Research University of Agder Kristiansand Norway

**Keywords:** Authorea, *bioRxiv*, career status, EcoEvoArxiv, preprint servers

## Abstract

The usage of preprint servers in ecology and evolution is increasing, allowing research to be rapidly disseminated and available through open access at no cost. Early Career Researchers (ECRs) often have limited experience with the peer review process, which can be challenging when trying to build publication records and demonstrate research ability for funding opportunities, scholarships, grants, or faculty positions. ECRs face different challenges relative to researchers with permanent positions and established research programs. These challenges might also vary according to institution size and country, which are factors associated with the availability of funding for open access journals. We predicted that the career stage and institution size impact the relative usage of preprint servers among researchers in ecology and evolution. Using data collected from 500 articles (100 from each of two open access journals, two closed access journals, and a preprint server), we showed that ECRs generated more preprints relative to non‐ECRs, for both first and last authors. We speculate that this pattern is reflective of the advantages of quick and open access research that is disproportionately beneficial to ECRs. There is also a marginal association between first author, institution size, and preprint usage, whereby the number of preprints tends to increase with institution size for ECRs. The United States and United Kingdom contributed the greatest number of preprints by ECRs, whereas non‐Western countries contributed relatively fewer preprints. This empirical evidence that preprint usage varies with the career stage, institution size, and country helps to identify barriers surrounding large‐scale adoption of preprinting in ecology and evolution.

## Graphical abstract

We predicted that the career stage and institution size would impact the relative usage of preprint servers among researchers in ecology and evolution. Using data collected from 500 articles, we demonstrate that ECRs generate more preprints relative to non‐ECRs, for both first and last authors. We speculate that this pattern is reflective of the advantages of quick and open access research that is disproportionately beneficial to ECRs.

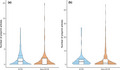

## 1 INTRODUCTION

Preprints are free, publicly accessible, early versions of research articles. They are posted online prior to, or in parallel with, the peer review process and helps shorten the temporal gap between completed studies and accessible research (Sarabipour et al., 2019; Vale, 2015). *BioRxiv* is one of the most popular preprint servers in the fields of ecology and evolution and hosts preprint articles with barrier‐free access to manuscripts (Hyland, 2016; Merga & Mason, 2020). Preprints usually appear on *bioRxiv* within 48 hr (https://www.biorxiv.org/about/FAQ), whereas manuscripts accepted in the first journal are submitted to take approximately 4 months to become visible (Himmelstein, 2015; Royle, 2015). It is not uncommon for manuscripts to be submitted consecutively to more than one journal, and, as a result, the peer review process can take years (Cobb, 2017). The usage of preprints is likely driven by open access research availability and recognition; it indicates that a paper is complete and ready for peer review. Preprints facilitate the sharing of knowledge prior to peer review and improve transparency through open access research. Increased availability and recognition of preprints has also led to increased citations (Serghiou & Ioannidis, 2018; Shuai et al., 2012), which is a key metric by which researchers are evaluated (Nicholas et al., 2019). Ultimately, preprints can be beneficial to all researchers, but the use of preprints might be especially beneficial to the unique challenges that Early Career Researchers (ECRs) face relative to senior researchers.

In this context, ECRs are defined as individuals who are at the beginning stages of their research careers and have not yet established research programs or gained tenured positions (Laudel & Gläser, 2008; Nicholas et al., 2019). This cohort includes undergraduate students, graduate students undertaking masters or doctoral degrees, postdoctoral researchers, and untenured professors and researchers. In contrast, senior researchers are individuals who have held independent academic positions for >5 years. While both groups hold research positions and are members of the scientific community, they face different challenges when publishing peer‐reviewed research (Laudel & Gläser, 2008). peer review is the process of subjecting an author's scholarly work, research, or ideas to the scrutiny of others who are experts in the field (Kelly et al., 2014). The validity of novel scientific research is evaluated prior to publication and dissemination. The peer review process requires that several relevant but impartial experts in the field closely examine the manuscript and determine its value to the scientific community. The value of peer review lies in its responsibility to determine the importance and originality of research, as well as identify any scientific or methodological errors. As such, the peer review process can be lengthy and even overwhelming for researchers, especially for ECRs who face compounding challenges due to their career stage.

The usage of preprints could provide some relief with respect to these challenges. Preprints can help counteract the collaborative, financial, and time constraints that ECRs face and provide an opportunity for them to gain feedback from peers in a cost‐effective manner (Merga & Mason, 2020). ECRs typically have less funding than established researchers. Therefore, ECRs may not be able to share their research in open access journals that allow for increased visibility and discussion of their research because they cannot afford the Article Processing Charges (APCs) that are integral to the typical open access publishing model (Merga & Mason, 2020). Publishing articles in open access journals often costs thousands of dollars in APCs, which is prohibitive for many ECRs lacking financial support, whereas the per‐paper processing costs of preprints are low and the cost to researchers is absent (Sarabipour et al., 2019). This lower cost makes preprints potentially vital to ECRs, as they provide opportunities to increase the volume and quality of free and informal feedback and collaboration on a greater scale relative to the typical peer review process that averages two formal reviews (Huisman & Smits, 2017; Penfold & Polka, 2020; Sarabipour et al., 2019). Additionally, the costs associated with open access publishing may be the reason that small institutions publish proportionately fewer open access articles (Shafer, 2020). These disparities emphasize the discriminatory nature of APCs against authors with little access to funding (Burchardt, 2014) and the disproportionate benefit to preprint usage among institutions of different sizes. Ultimately, this literature highlights the financial burden of publishing research in open access journals and provides further motivation for the use of a low‐cost preprint journal.

There is some resistance to preprinting, especially as perceived by senior researchers, as no formal peer review takes place and the onus is on the reader to interpret the accuracy and significance of the findings (Bove‐Fenderson et al., 2018; Fry et al., 2019). However, making research readily available while it undergoes peer review facilitates early public access to novel data and methodologies that can inform other ECRs' decisions regarding their own research, saving time and money. Employment in the fields of ecology and evolution often requires quality scholarly research outputs for career advancement, which can be a challenge for ECRs (Hyland, 2016; Merga & Mason, 2020). While an excellent publication record is important at all career stages, the increased stakes due to lower levels of job security for ECRs and the typically limited time window for applying for scholarships, grants, or faculty positions make this delay between submission and publication particularly detrimental for individuals at this stage. Preprints can reduce these barriers by allowing ECRs to make their work publicly available more rapidly and at no cost, thus increasing research visibility (Serghiou & Ioannidis, 2018) and ultimately assisting in career development (Berg et al., 2016).

As masters and PhD programs are often short term (typically 2–5 years; DeClou, 2017), there is increased pressure on graduate students to conduct and publish high quality research in a short time frame (Browning et al., 2017). ECRs also face challenges surrounding financial and employment instability (DeClou, 2017; Nicholas et al., 2017). These challenges are faced less often by senior researchers, who typically have tenure or positions of power at their organization (McAlpine & Amundsen, 2015). The challenges of writing, managing journal requirements, and dealing with the peer review process are shared among all researchers. However, the experience is likely different between ECRs and non‐ECRs, as the familiarity with peer review and publication differs (Nicholas et al., 2017). As ECRs are still developing their research niche, they often have limited support in the form of experienced colleagues and funding, both of which researchers tend to gain over time (Bazeley, 2003; McAlpine & Amundsen, 2015). Concurrently, there is an increasing demand for research that leads to social, economic, and policy change (Chikoore et al., 2016). Translating research into these fields takes additional time. Therefore, time limited ECRs are disproportionately affected by these demands. However, preprints can potentially help to address some of these issues.

The discussion regarding the benefit of increased preprint prevalence is ongoing. Of note, we opted to have *Ecology and Evolution* facilitate the preprinting of our journal submission using the preprint server Authorea. Among others, the journal eLife proposes a shift from the typical peer review process to a more open and transparent framework, where journals help transform preprints into high‐quality published manuscripts (Eisen et al., 2020). We shed light on some of the factors involved in such a transition.

Herein, we examine articles in four popular journals in the fields of ecology and evolution and one preprint server to assess whether the usage of preprints differs based on career stage. Due to the challenges that ECRs face and the benefits that preprints might provide them, we predict that ECRs disproportionately utilize preprint servers relative to senior researchers. Specifically, we predict that, when either the first or last author was identified as an ECR, it would have a positive association with the number of preprints by the first author. We also explored the association between institution size and preprint rates because of the positive association of institution size with funding availability and open access publication rates (Shafer, 2020). We additionally examined potential differences between countries based on well‐documented geographic financial and cultural disparities (Abdill et al., 2020; Robinson‐Garcia et al., 2020).

## 2 METHODS

### 2.1 Data collection

Articles published in 2019 from two open access journals (*Ecology and Evolution* and *PLOS One*), two subscription‐based journals (*Proceedings of the Royal Society B* and *Ecology*), and one preprint server (*bioRxiv*) were selected using the search term ‘ecology and evolution’ in each journal's webpage. The first 100 full‐text English articles from each journal were collected to produce a final dataset of 200 open access articles, 200 closed access articles, and 100 preprints. Fewer than 100 articles from *Proceedings of the Royal Society B* in 2019 fit our criteria, so the search was expanded to include publications in 2020 to achieve the desired sample size. The title of the article, number of authors, names of first and last authors, and author affiliations were collected. The total numbers of publications for first and last authors were determined using authors' Google Scholar profiles. Articles were excluded if the first or last author did not have a Google Scholar profile. By excluding the work of authors that do not have a Google Scholar profile, certain career stages may have been disproportionately excluded depending on the relationship between career stage and Google Scholar profile usage. Removing data disproportionately from certain career stages could have resulted in null or unclear relationships. However, we felt that our sample sizes across career stages were sufficient. The *bioRxiv* database was used to determine the number of preprints an author has submitted. We defined ECR as a student at any stage (Laudel & Gläser, 2008), as well as individuals that held an independent academic position for 5 years or less (NSERC, 2020). The ECR status of the first and last authors were determined by examining institutional profiles and websites, Google Scholar, or personal websites, in that order. Articles were excluded if the ECR status could not be determined. Lastly, information on the affiliations of the first and last authors, including size and country were collected. Institutions were quantified as small (<10,000 students), medium (10,001–19,999 students), or large (>20,000 students), following Shafer (2020).

### 2.2 Data analysis

The base package in R v4.0.2 was used for statistical modelling (R Core Team, 2020). A generalized linear model (GLM) was run, with the number of preprint articles as the response variable. Fixed explanatory variables included the career status of both the first and last authors (two‐level factors) and the institution size of the first author (three‐level factor). The total number of publications by the first author was log transformed and included as an offset variable, so that we were able to effectively model the relationship as a rate and use the Poisson distribution (Shafer, 2020). A Poisson GLM indicated that there was overdispersion in the data. We corrected the standard errors using a quasi‐GLM model where the variance is given by Ψ × µ, µ is the mean, and Ψ is the dispersion parameter (Zuur et al., 2019).

To determine if an individual who publishes more and generates more preprints, we tested for a correlation between the number of preprints and total publications of first and last authors using a Spearman's rank correlation test. Furthermore, we completed a one‐way analysis of variance (ANOVA) to determine if the mean number of preprints differed by the country associated with the first author. We then used ArcGIS Pro (version 2.6.3) to visualize the distribution of preprints and proportion of early career researchers by country. The percent of total preprints represents the sum of first author preprints in each country divided by the total number of preprints counted in this study. The proportion ECR represents the proportion of preprints in each country with an ECR as the first author. A value of 1 indicates all preprints were submitted by an ECR and a value of 0 indicates that all preprints were submitted by a non‐ECR.

## 3 RESULTS

**FIGURE 1:**
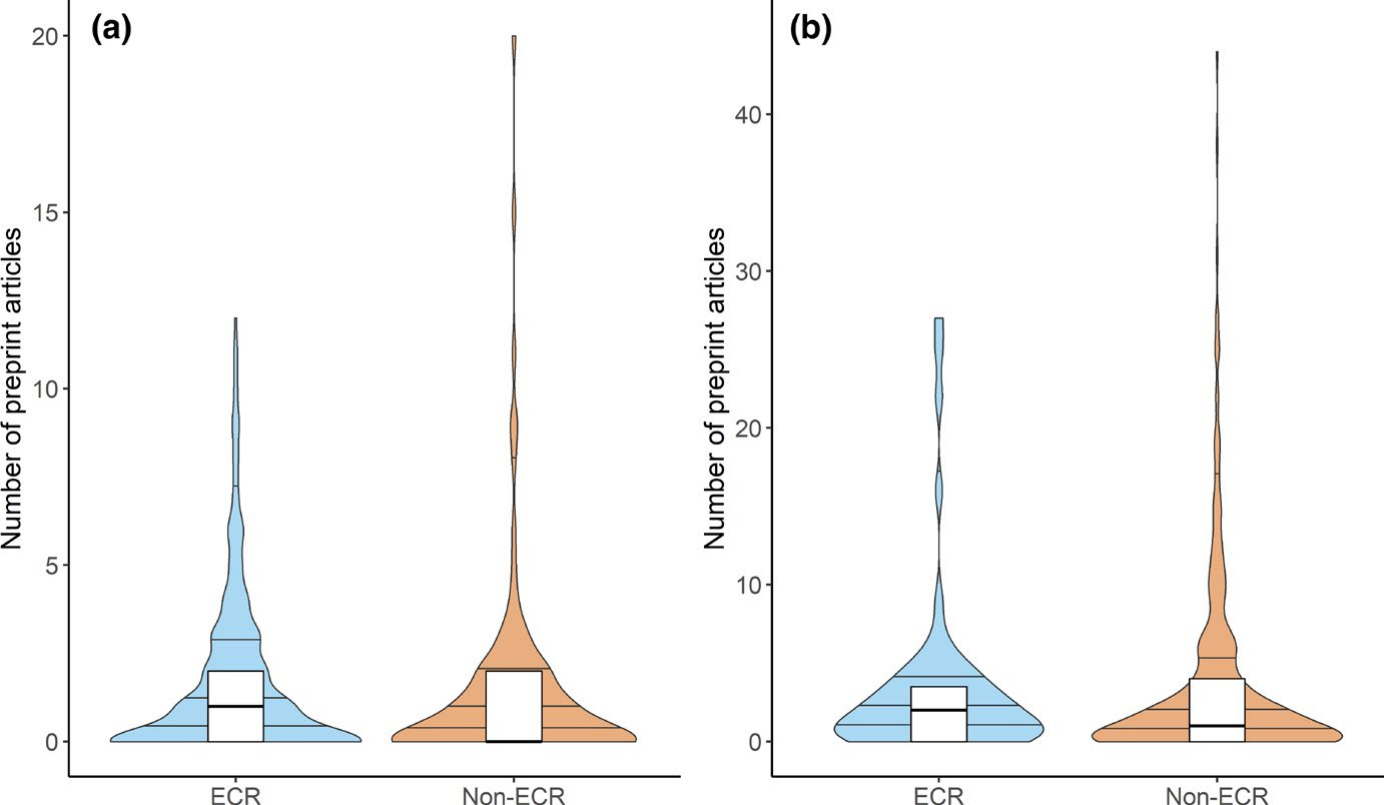
The raw numbers of preprint articles produced by (a) first and (b) last authors of ecology and evolution articles (*n* = 500) according to Early Career Researcher (ECR) status. Lines in the violins represent the 25%, 50%, 75%, and 95% quartiles

A total of 500 articles were included in our analysis (*Proceedings of the Royal Society B* = 100 [*n*_2019_ = 49, *n*_2020_ = 51], *PLoS ONE* = 100, *Ecology* = 100; *Ecology and Evolution* = 100, *bioRxiv* = 100). The quasi‐GLM indicated that career status of both first and last authors had significant associations with the number of preprints generated by the first author (Figure 1a,b, Tables 1 and 2). Within the 500 articles, when either the first or last author was not an ECR, there were relatively fewer preprints generated (Figure 1); however, this association was greater when the first author was not an ECR (Table 2). There is also a marginal association between first author institution size and preprint usage, whereby the number of preprints tends to increase with institution size (Table 2). Ultimately, this indicates that ECRs generate more preprints relative to non‐ECRs, with institution size also impacting this relationship.

**TABLE 1:** Deviance table from generalized linear models for the associations between first author Early Career Researcher (ECR) status, last author ECR status, and first author institution size on the number of preprints generated by the first author

Model term	*df*	Deviance	Residual *df*	Residual deviance	*p*‐value
Null			498	2,102.5	
First author ECR status	1	416.94	497	1,685.5	**<2.2** × **10^–16^**
Last author ECR status	1	28.319	496	1,657.2	**.002**
First author institution size	2	29.538	494	1,627.7	.057

Note

The *p*‐values were obtained from *χ* ^2^ tests of whether the model fit improved by sequentially adding first author ECR status, last author ECR status, and first author institution size to the null model. Significance values are in bold and were determined using *α* = 0.05.

**TABLE 2:** Results from a quasipoisson family generalized linear model, where the number of preprint articles of the first author is the response variable

Incidence rate ratios		Confidence interval	*p*‐value
(Intercept)	0.12	0.08–0.17	**<.001**
First author status: non‐early career researcher	0.1	0.14–0.31	**<.001**
Last author status: non‐early career researcher	0.62	0.42–0.94	**.020**
Medium‐sized institution	0.86	0.54–1.33	.53
Small‐sized institution	0.44	0.18–0.90	**.045**

Note

Significance values are in bold and were measured using *α* = 0.05. The intercept in this model represents Early Career Researchers (ECRs) at large institutions.

**FIGURE 2:**
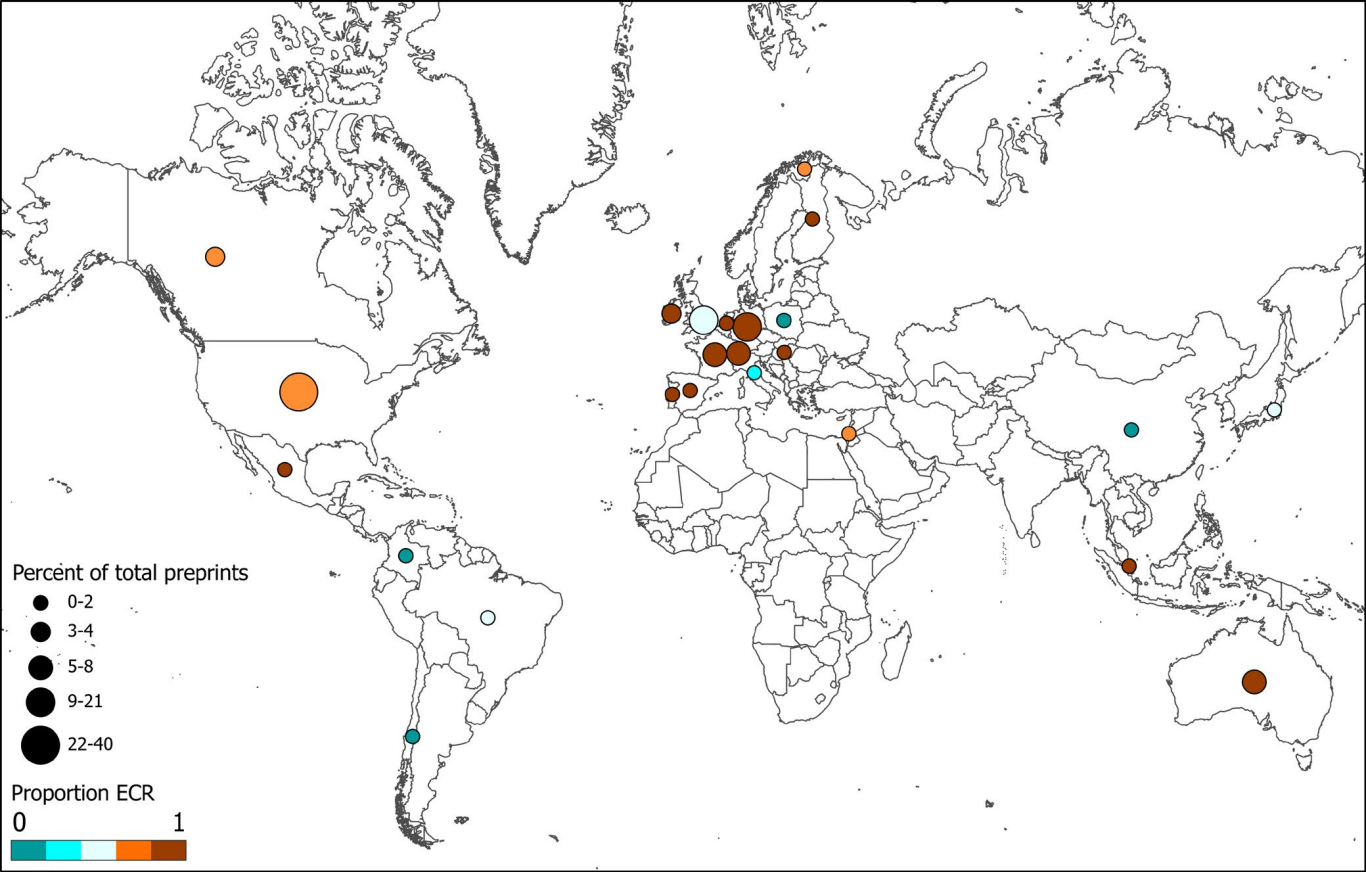
Geographical distribution of the percentage of preprints (*n* = 734) generated by first and last authors of 500 recent (2019–2020) ecology and evolution articles. Color represents the proportion of Early Career Researcher (ECR) authors by country

Spearman's rank correlation tests indicated no correlation between the number of preprints and total number of publications for the first author (*r* = 0.031, *p* = .50) or last author (*r* = 0.050, *p* = .28). When considering the totality of preprints generated by first and last authors from the 500 articles (*n* = 734), the United States and the United Kingdom contributed the greatest number of preprints by early career researchers, whereas non‐Western countries contributed fewer preprints (Figure 2). However, there was no significant difference in the mean number of preprints of the first author between countries (*F*_39,401_ = 1.177, *p* = .22).

## 4 DISCUSSION

Preprints are used differentially by researchers at different career stages and vary with institution size. ECRs generate more preprints than non‐ECRs and preprint usage tends to be greater among first authors at large institutions. Therefore, ECRs at large institutions typically generate the most preprints, while non‐ECRs at small institutions tend to generate the fewest preprints. Additionally, certain western countries like the United Kingdom and the United States contribute more preprints than others. Preprints are already benefiting ECRs and the broader scientific community (Penfold & Polka, 2020), but the large‐scale adoption of preprints across all career stages and institution sizes is necessary for these benefits to be distributed equitably. A strong preprint culture has the potential to reduce the negative impacts of the current publishing landscape with respect to the lengthy review process and financial burdens associated with open access research, which disproportionately impact ECRs. Increased preprint usage within the scientific community, in general, will help to create a more equitable environment for both ECRs and non‐ECRs.

The usage of preprint servers has many advantages for ECRs, including the rapid dissemination of research, broad visibility of research output through open access, and more inclusive and transparent peer review (Desjardins‐Proulx et al., 2013; Penfold & Polka, 2020; Sarabipour et al., 2019). Developing an environment where preprinting is the norm allows individuals to disseminate their research and build a reputation while increasing the likelihood that their work will be recognized by grant or job evaluation committees (Desjardins‐Proulx et al., 2013).

Senior authors often play a crucial role with respect to an ECR's opportunity to publish in a journal with a moderate‐to‐high impact factor (Sekara et al., 2018). Preprints offer a space that is free of publication bias and encourages sharing of diverse researchers' works, at any career stage (Jennions & Møller, 2002; Sarabipour et al., 2019). However, ECRs typically have less experience, influence, and security relative to senior researchers, thereby exerting less control over the decision to utilize a preprint server. As such, the balance of power often lies with the senior researcher, wherein they may not support an ECR's choice to utilize a preprint server. It is possible that the senior researcher may perceive preprinting to offer no benefit or even cause harm to themselves, their trainee(s), or the field at large. For example, perceived concerns about being ‘scooped’ (i.e., competing researchers using knowledge gained from the preprint to publish similar findings in the peer‐reviewed literature before the preprint authors) have been raised, though evidence is lacking to support this as a substantial risk (Penfold & Polka, 2020; Sever et al., 2019; Sever et al., 2019). Related to this are perceived concerns that preprints, although assigned a digital object identifier (DOI) establishing a permanent record of submission date and content, will not be sufficient for establishing precedence of discoveries (Sever et al., 2019; Sever et al., 2019). To our knowledge, this has not been widely tested. Another perceived concern is that preprint servers provide a means of widely disseminating poor quality research, which can then be cited, thus undermining the integrity of the scientific literature (Maggio et al., 2018). However, similar concerns exist for peer‐reviewed journal articles (e.g., Wakefield et al., 1998). Additionally, two‐thirds of preprints posted on *bioRxiv* before 2017 were later published in peer‐reviewed journals, indicating that most preprints are ultimately published in the peer‐reviewed literature (Abdill & Blekhman, 2019). Senior researchers might choose to place these perceived concerns above the potential benefits for ECRs. However, the interplay between ECRs and senior researchers regarding the usage of preprint servers is likely complex. ECRs might also be skeptical to use preprint servers if they are insecure about their research being made public prior to being vetted by impartial experts. This perceived concern might be especially prevalent when ECRs engage with a new subject, stray from the research area of their advisor(s), or belong to underrepresented or marginalized groups that disproportionately suffer from “imposter syndrome” in academia (Bravata et al., 2020; Pulliam & Gonzalez, 2018). Preprint mandates have been suggested to mitigate some of these issues. For example, in Plan U, funding agencies would mandate the posting of preprints by grantees (Server et al., 2019). This might help to alleviate concerns regarding whether to use preprint servers or not, as senior researchers and ECRs alike would be required to preprint. Mandating preprints could also improve preprint quality, however, future research is needed in this area. While we show that the ECR status of first and last authors influences preprint usage, the decision‐making process behind this trend should be explored in future research.

Smaller institutions publish fewer open access articles relative to researchers at large universities (Shafer, 2020), a trend that seems to hold true for preprints despite their accessibility (Figure 1; Table 2). Further, data from Robinson‐Garcia et al., (2020) suggests that countries with higher rates of open access publications than the median also exhibit greater rates of preprint usage. This might be detrimental for ECRs at smaller institutions, as it could further limit their research impact and could place ECRs at small universities in countries with low open access rates at an even greater disadvantage. Further, our results uncovered geographical heterogeneity in the rate of preprint usage (Figure 2). South American and Asian countries published fewer preprints than North American and European countries as well as Australia (Figure 2). Countries with some of the highest open access publications rates (e.g., United Kingdom, United States, France, and Spain; Robinson‐Garcia et al., 2020) also produced some of the highest rates of preprint usage, consistent with the findings of Abdill et al., (2020). Countries with lower open access publication rates generally exhibited lower preprint usage rates. An exception to this is Australia, which contributed a high number of preprints while posting an open access publication rate slightly below the global median (Robinson‐Garcia et al., 2020). Our results indicate that preprint publications within a country mostly mimic that country's open access publication landscape. Preprint mandates might be beneficial here, as they would facilitate free access to research articles worldwide with minimal effort.

Although not examined directly in the present study, the potential conflicts and disparities surrounding preprinting are likely further compounded among ECRs who belong to underrepresented groups. Underrepresented and historically marginalized groups face additional barriers to advancing in the fields of ecology and evolution (Fox et al., 2018, Fox et al., 2019; Fox & Paine, 2019; Miriti et al., 2020; Schell et al., 2020; Tseng et al., 2020). In particular, women and Black, Indigenous, and People of Color (BIPOC) are underrepresented in ecology and evolution, especially in positions of power (O'Brien, Bart, & Garcia,  2020; Wei et al., 2020). In the United States, between 2014 and 2018, only 1.2% of ecology and evolutionary biology PhD graduates identified as Black or Indigenous. This contrasts with the 16% of the American population identifying with those demographics (Tseng et al., 2020). The lack of ethnic diversity in ecology and evolution can be attributed to a variety of factors including systemic disparities in education between communities and a lack of role models leading to a reduced sense of belonging in the field for non‐white students (O'Brien et al., 2020). Due to the discrepancies between the general population and demographics of the field, it would be remiss to assume that all ECRs face the same challenges. The accumulation of challenges due to the intersectionality of career status and other social identities further amplifies the difficulties that many individuals experience (Crenshaw, 1989; Wanelik et al., 2020). There are barriers at every career stage and the disparity between ECRs and non‐ECRs in preprint usage potentially exacerbates the difficulties that underrepresented ECRs face, which is a topic that warrants further study.

In addition to the challenges faced by ethnic minorities, women are also underrepresented in this field. In ecology and zoology, women represent less than one third of authors and research groups led by men published with <20% female coauthors, whereas female‐led groups published with >60% female coauthors (Salerno et al., 2019). We initially considered exploring the relationship between ECR status and preprint usage among individuals of different genders within this study. However, without data on self‐reported gender identities, we were unable to do so in a way that does not risk misidentifying and causing harm to individuals (e.g., by using gender assignment tools based on first names; Cameron & Stinson, 2019). While gender is an important factor to analyze in respect to research trends in ecology and evolution, until there is a method by which individuals can self‐identify their preferred gender/pronouns, we do not feel it is appropriate to use tools that may misassign gender and cause harm. Subsequently, we suggest that this provides motivation for authors to include their self‐identified pronouns in research profiles, biographies, and publications (as we have done in the present work). Perhaps ideally, such demographic information would be collected safely during the submission process to enhance standardization for downstream analyses. These actions might enable publication trends of underrepresented groups to be explored whilst ensuring the safety of authors. However, such a process should be implemented in consultation with an expert in diversity, equity, and inclusion issues to ensure that potential negative indirect effects are avoided.

## 5 CONCLUSION

This study indicates that preprint servers are used disproportionately between ECRs and non‐ECRs at institutions of different sizes. We suggest key factors that may lead to the differential usage of preprints among researchers of varying career stages and discuss the effects that using preprint servers can have on career development. We note that there are myriad benefits to using preprint servers that are specifically valuable for ECRs, which may explain the greater preprint usage in this group. Open access research is associated with increased citations, media attention, potential collaborations, and job and funding opportunities (Fernandes et al., 2020; Fu & Hughey, 2019; McKiernan et al., 2016). These benefits might drive ECRs to make their research publicly accessible and, in turn, be linked to the greater usage of preprint servers by ECRs.

This research provides evidence for the unequal usage of preprint servers among researchers of varying career stages and is necessary to facilitate further discussion surrounding the larger‐scale adoption of preprints in the field of ecology and evolution. There are ongoing discussions regarding the adoption of mandatory usage of preprint servers prior to peer review (Desjardins‐Proulx et al., 2013). It is evident that career stage influences preprint usage, and due to the multitude of benefits, we believe further discussions and studies of this kind are necessary to address the unique needs of ECRs with respect to preprint usage. This study aims to illuminate the landscape of preprint servers specifically in ecology and evolution, whereas future research should aim to determine the cause(s) of disproportionate usage of preprints by ECRs at larger institutions to help reduce possible barriers to preprinting for other groups. Ultimately, a strong and widely adopted preprint culture in ecology and evolution may help facilitate greater preprint usage among historically marginalized groups, aiding in career development for those who are underrepresented in the field.

## CONFLICT OF INTEREST

The authors declare that they have no conflicts of interest.

## AUTHOR CONTRIBUTIONS

**Jesse F. Wolf:** Conceptualization (lead); Data curation (lead); Formal analysis (lead); Investigation (equal); Methodology (lead); Project administration (lead); Visualization (lead); Writing‐original draft (equal); Writing‐review & editing (equal). **Layla MacKay:** Conceptualization (equal); Data curation (equal); Formal analysis (equal); Investigation (equal); Methodology (equal); Writing‐original draft (equal); Writing‐review & editing (equal). **Sarah E. Haworth:** Conceptualization (equal); Data curation (equal); Formal analysis (equal); Investigation (equal); Methodology (equal); Writing‐original draft (equal); Writing‐review & editing (equal). **Marie‐Laurence Cossette:** Conceptualization (equal); Data curation (equal); Formal analysis (equal); Investigation (equal); Methodology (equal); Writing‐original draft (equal); Writing‐review & editing (equal). **Morgan N. Dedato:** Conceptualization (equal); Data curation (equal); Formal analysis (equal); Investigation (equal); Methodology (equal); Writing‐original draft (equal); Writing‐review & editing (equal). **Kiana B. Young:** Conceptualization (equal); Data curation (equal); Formal analysis (equal); Investigation (equal); Methodology (equal); Visualization (lead); Writing‐original draft (equal); Writing‐review & editing (equal). **Colin I. Elliott:** Conceptualization (equal); Data curation (equal); Formal analysis (equal); Investigation (equal); Methodology (equal); Writing‐original draft (equal); Writing‐review & editing (equal). **Rebekah A. Oomen:** Formal analysis (supporting); Investigation (equal); Methodology (supporting); Supervision (lead); Visualization (supporting); Writing‐review & editing (equal).

## Data Availability

The raw data used to perform analyses and generate figures for this manuscript are available at https://doi.org/10.5061/dryad.wstqjq2n3.
